# Supplementary material for: Splice-Junction-Based Mapping of Alternative Isoforms in the Human Proteome
Source: Cell Rep. Author manuscript; Available in PMC 2020 Jan 15. (PMC6961840; doi:10.1016/j.celrep.2019.11.026)
Supplement: 3 [file NIHMS1546469-supplement-3.zip › DF2/PXD0010154/Lung-182-Q9NS15-QCLSPEEMER.pdf]

A

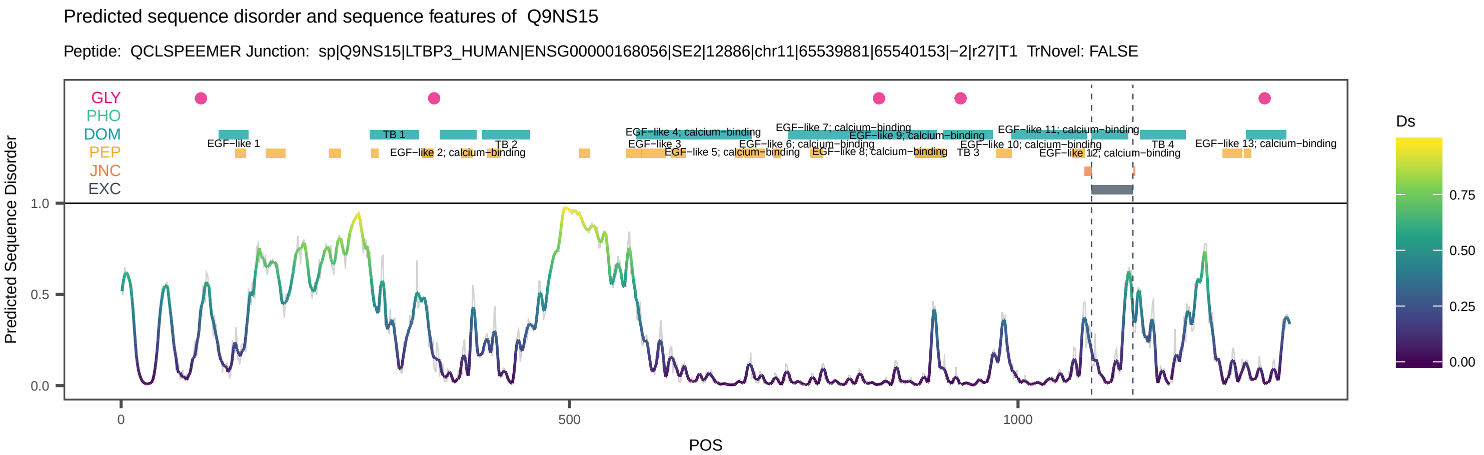

Predicted sequence disorder and sequence features of Q9NS15
Peptide: QCLSPEEMER Junction: sp|Q9NS15|LTBP3_HUMAN|ENSG00000168056|SE2|12886|chr11|65539881|65540153|−2|r27|T1 TrNovel: FALSE
GLY
PHO
DOM
PEP
JNC
EXC
Predicted Sequence Disorder
POS
Ds

B

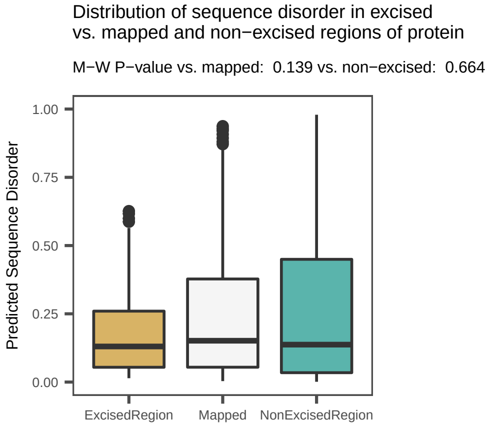

Distribution of sequence disorder in excised vs. mapped and non−excised regions of protein
M−W P−value vs. mapped: 0.139 vs. non−excised: 0.664
Predicted Sequence Disorder
ExcisedRegion
Mapped
NonExcisedRegion

C
